# T-independent antigen induces humoral memory through germinal centers

**DOI:** 10.1084/jem.20210527

**Published:** 2022-01-12

**Authors:** Xin Liu, Yongshan Zhao, Hai Qi

**Affiliations:** 1 Tsinghua-Peking Center for Life Sciences, Beijing, China; 2 Laboratory of Dynamic Immunobiology, Institute for Immunology, Tsinghua University, Beijing, China; 3 Department of Basic Medical Sciences, School of Medicine, Tsinghua University, Beijing, China; 4 Beijing Key Laboratory for Immunological Research on Chronic Diseases, Tsinghua University, Beijing, China; 5 Beijing Frontier Research Center for Biological Structure, Tsinghua University, Beijing, China

## Abstract

T-dependent humoral responses generate long-lived memory B cells and plasma cells (PCs) predominantly through germinal center (GC) reaction. In human and mouse, memory B cells and long-lived PCs are also generated during immune responses to T-independent antigen, including bacterial polysaccharides, although the underlying mechanism for such T-independent humoral memory is not clear. While T-independent antigen can induce GCs, they are transient and thought to be nonproductive. Unexpectedly, by genetic fate-mapping, we find that these GCs actually output memory B cells and PCs. Using a conditional BCL6 deletion approach, we show memory B cells and PCs fail to last when T-independent GCs are precluded, suggesting that the GC experience per se is important for programming longevity of T-independent memory B cells and PCs. Consistent with the fact that infants cannot mount long-lived humoral memory to T-independent antigen, B cells from young animals intrinsically fail to form T-independent GCs. Our results suggest that T-independent GCs support humoral memory, and GC induction may be key to effective vaccines with T-independent antigen.

## Teaser

T-independent antigens are known to induce transient germinal center formation. Liu et al. show that these germinal centers output memory B cells and plasma cells and condition their persistence.

## Introduction

Type II T-independent (TI-II) antigens contain highly repetitive structures and are able to stimulate B cells to proliferate and differentiate into plasma cells (PCs) and memory B (B_mem_) cells without classic cognate T cell help. However, in most cases, responses induced by these antigens are extrafollicular and short-lived, subsiding within days (Nutt et al., 2015). Some TI-II antigens are known to induce the formation of long-lived PCs and B_mem_ cells in both mice and humans (Bortnick and Allman, 2013) and are the antigen components in widely used vaccines (Robbins et al., 1983). These vaccines can be produced at a relatively low cost and are important weapons in the fight against encapsulated bacterial infections, which cause more a million deaths worldwide each year (Watt et al., 2009, GBD 2016 Lower Respiratory Infections Collaborators, 2018, GBD 2016 Meningitis Collaborators, 2018). However, challenges remain in the design of polysaccharide vaccines due to incomplete understanding of how they function. For example, polysaccharide vaccines do not induce consistent responses in infants, leaving the most susceptible population unprotected (Rijkers et al., 1998; Robbins et al., 1983). Therefore, it is important to understand the mechanisms of how TI antigens induce immune memory formation.

Long-lived PCs and B_mem_ cells are derived from germinal centers (GCs) in T-dependent (TD) responses elicited by protein antigens. Within GCs, B cells undergo somatic hypermutations of their B cell receptors, and high affinity clones are selected by follicular helper T cells for greater expansion, as well as differentiation into B_mem_ cells and long-lived PCs (Victora and Nussenzweig, 2012). Some polysaccharide antigens are also known to stimulate GC formation without involving T cells (de Vinuesa et al., 2000; Lentz and Manser, 2001). These TI GC B cells bear molecular signatures very similar to their TD counterparts (Yu et al., 2008), although they can last for only a few days (de Vinuesa et al., 2000; Lentz and Manser, 2001). It is unclear whether such TI GCs can produce any B_mem_ cells or PCs.

Here we analyzed cellular outputs from TI GCs and found that TI GCs produces both B_mem_ cells and PCs, and that the GC experience promotes the persistence of these output cells. Furthermore, in contrast to adult B cells, infant B cells are intrinsically less capable of forming TI GCs.

## Results and discussion

### TI GCs collapse without extensive apoptosis

**Figure 1.**
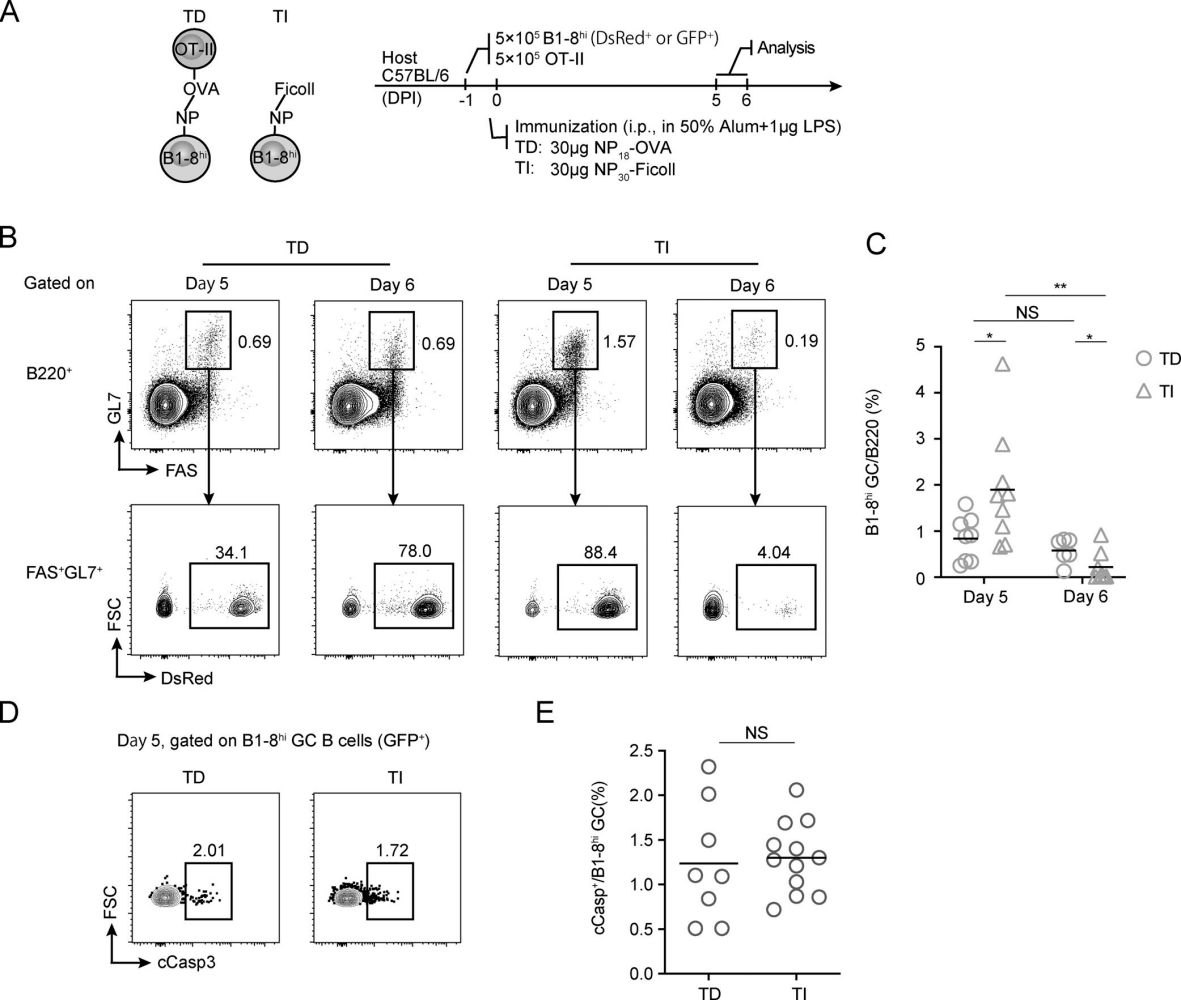
**TI GCs collapse without extensive apoptosis. (A)** The principle (left) and protocols (right) for constructing TD and TI GCs. **(B and C)** Abundance of TI and TD GCs. **(B)** Representative FACS profiles for gating of B1-8^hi^ TD and TI GCs at indicated time after immunization. **(C)** Summary statistics of B1-8^hi^ GC% in B cells. **(D and E)** Apoptosis in TD and TI GCs. **(D)** Representative FACS plots of cCasp staining of B1-8^hi^ GC B cells. **(E)** Summary statistics of cCasp^+^% in B1-8^hi^ GCs. Data were pooled from two (B and C) or three (D and E) independent experiments with at least two mice per group. Each symbol indicates one mouse, and lines denote means. P values by Student’s *t* test. *, P < 0.05; **, P < 0.01. DPI, days postimmunization; FSC, forward scatter.

**Figure S1.**
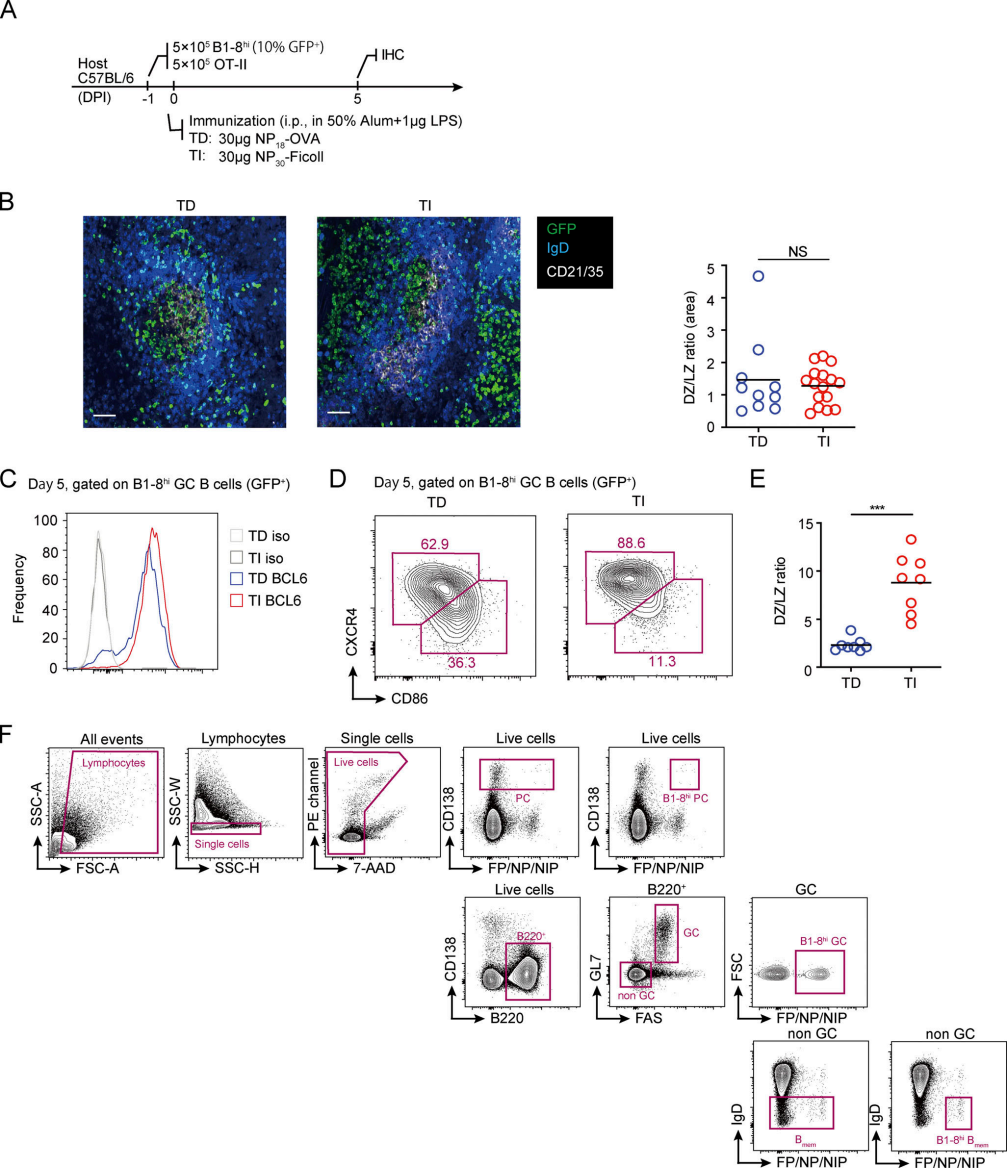
**DZ-LZ polarization in TI GCs. (A)** Experimental outline. TD and TI GCs were constructed as described in Fig. 1. **(B)** Immunohistochemical staining of spleen sections from mice bearing TD and TI GCs, respectively (left). The ratio between the area occupied by DZ and LZ was calculated (right). The GC area was defined by clearance of IgD^+^ naive follicular B cells, and the LZ was defined by the presence of CD21^+^ follicular dendritic cells. Scale bar denotes 100 µm. Data were representative of two independent experiments, with at least two mice analyzed in each group. Each symbol indicates one GC, and lines denote means. **(C)** FACS analysis of BCL6 expression levels in TD and TI B1-8^hi^ GC B cells. **(D and E)** FACS analysis of DZ and LZ phenotype cells within B1-8^hi^ GCs. **(D)** FACS plots showing gatings of DZ and LZ cells in TD and TI GCs, respectively. DZ cells were gated as CXCR4^hi^CD86^lo^, and LZ cells were gated as CXCR4^lo^CD86^hi^. **(E)** Quantification of DZ/LZ ratio in B1-8^hi^ TD and TI GCs based on data in B. Data were pooled from three independent experiments with at least two mice per group. Each symbol indicates one mouse, and lines denote means. **(F)** Gating strategies for B1-8^hi^ GC B cells, B_mem_ cells, and PCs. P values by Mann–Whitney test. ***, P < 0.001.

TI-II antigens, such as 4-hydroxy-3-nitrophenylacetic (NP)-Ficoll and dextran, are able to induce GC formation in experimental animals (Lentz and Manser, 2001; Wang et al., 1994). However, when induced in a polyclonal background, TI GCs are formed only stochastically and usually not at a scale comparable to TD GCs (Lentz and Manser, 2001). TI GC formation can be modeled by adoptive transfer of antigen-specific naive B cells before immunization with TI antigens (de Vinuesa et al., 2000). To analyze potential output from TI GCs, we first established an adoptive transfer model that can robustly stimulate the formation of TI GCs and the control TD GCs in vivo (Fig. 1 A). In brief, 5 × 10^5^ DsRed-expressing B1-8^hi^ B cells bearing the B cell receptor specific for the hapten NP were transferred into C57BL/6 mice together with 5 × 10^5^ OT-II T cells, followed by i.p. immunization with NP_30_-Ficoll or NP_18_-OVA to stimulate TI and TD GC formation, respectively (Fig. 1 A). 5 d after immunization, transferred B1-8^hi^ GC B cells were detected in mice immunized with either of the immunogens by the expression of DsRed (Fig. 1 B). TI GCs reached an even higher level of abundance than did TD GCs on day 5 after immunization, but they were not well sustained, with most disappearing by day 6; on the other hand, TD GCs persisted as expected (Fig. 1, B and C). The apparent collapse of TI GCs confirms what has been reported previously (de Vinuesa et al., 2000). Before their rapid dissipation, TI GCs were morphologically indistinguishable from control TD GCs, being apparently partitioned by the follicular dendritic cell network into dark zones (DZs) and light zones (LZs) on tissue sections (Fig. S1, A and B). In addition, TI GC B cells highly express BCL6, comparable to the level found in TD GC B cells (Fig. S1 C). However, by flow cytometry, most TI GC cells did not actually show molecular markers of LZ B cells (Fig. S1, D and E), suggesting that full LZ-DZ polarization of GCs requires T cell participation (Victora et al., 2010).

**Figure 2.**
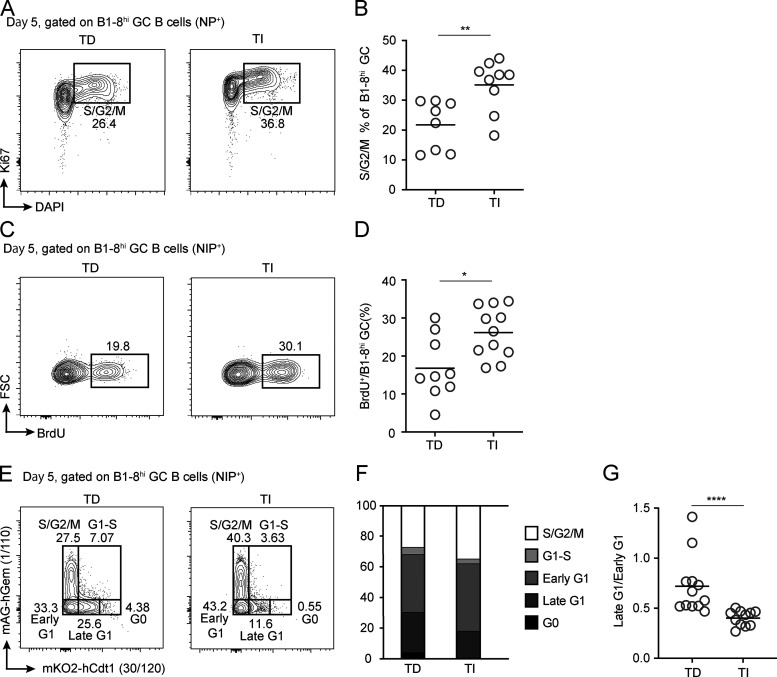
**Cell cycle analyses of TD and TI GCs. (A and B)** DNA contents and Ki67 expression of B1-8^hi^ TD and TI GC B cells. Representative FACS profiles (A) and summary statistics of cells in S/G2/M phase of cell cycle (B). **(C and D)** Acute BrdU incorporation in B1-8^hi^ TD and TI GC B cells. Representative FACS profiles (C) and summary statistics of B1-8^hi^ GC B cells incorporating BrdU after 30-min pulse (D). **(E–G)** TD and TI GCs analyzed with the FUCCI cell cycle reporter. **(E)** FACS plots showing cell cycle profiles of B1-8^hi^ TD and TI GC B cells, respectively, as enabled by the expression of the FUCCI reporter. **(F)** Percentages of B1-8^hi^ TD and TI GC B cells in different cell cycle stages. G0, mKO2^hi^; S/G2/M, mAG^+^; G1–S, mAG^+^mKO2^+^; early G1, mAG^−^mKO2^−^; late G1, mKO2^lo^. **(G**) The ratio of late G1 to early G1 cells in B1-8^hi^ TD and TI GCs. All summary data were pooled from three independent experiments with at least two mice per group. In scatter plots, each symbol indicates one mouse, and lines denote means. P values by Student’s *t* test (B and D) or Mann–Whitney *U* test (G). *, P < 0.05; **, P < 0.01; ****, P < 0.0001.

The sudden collapse of TI GCs could be due to massive death, even though they appeared to have grown from the same precursor frequency to a larger population 5 d after immunization (Fig. 1, B and C). To test this idea, we measured in GC B cells cleaved caspase 3 (cCasp3), which indicates commitment to death. Surprisingly, similar fractions of TI or TD GC B cells were cCasp3^+^ (Fig. 1, D and E), arguing against collective death as the reason for collapse. Alternatively, the sudden collapse of TI GCs could result from a complete stop of cell cycling. To test this idea, we took three independent approaches. First, we characterized cell cycle profiles of TI and TD GC B cells by measuring their DNA contents. Compared with TD GCs, there were actually more B cells at the S/G2/M phase but fewer cells at the G0 phase in TI GCs (Fig. 2, A and B), suggesting that they did not stop cycling but proliferated more actively. Second, when pulsed with BrdU for 30 min in vivo, a significantly higher percentage of TI GC B cells were BrdU^+^, indicating active DNA synthesis at the S-phase (Fig. 2, C and D). Finally, we also took advantage of B1-8^hi^ cells expressing a modified fluorescent ubiquitination-based cell cycle indicator (FUCCI) from the UBP-2A-FUCCI reporter mouse line (Wang et al., 2017). As previously described, the intensity of mKO2 fluorescence correlates with the duration of a cell having been in the G1 phase (Sakaue-Sawano et al., 2008; Wang et al., 2017). As shown in Fig. 2, E–G, when compared with TD GCs induced with UBP-2A-FUCCI B1-8^hi^ cells, the TI GC counterpart actually contained significantly fewer mKO2^+^ cells and essentially no mKO2^hi^ cells. Therefore, TI GC B cells did not stop cycling but actually spent less time in the G1 phase and made faster G1-to-S transition. Taken together, these results demonstrate that the rapid collapse of TI GCs is not due to collective apoptosis or reduced proliferation of B cells. The remaining scenario for the TI GC collapse is that most of cells therein are exported, possibly as B_mem_ cells and PCs.

### TI GC outputs both B_mem_ cells and PCs

**Figure 3.**
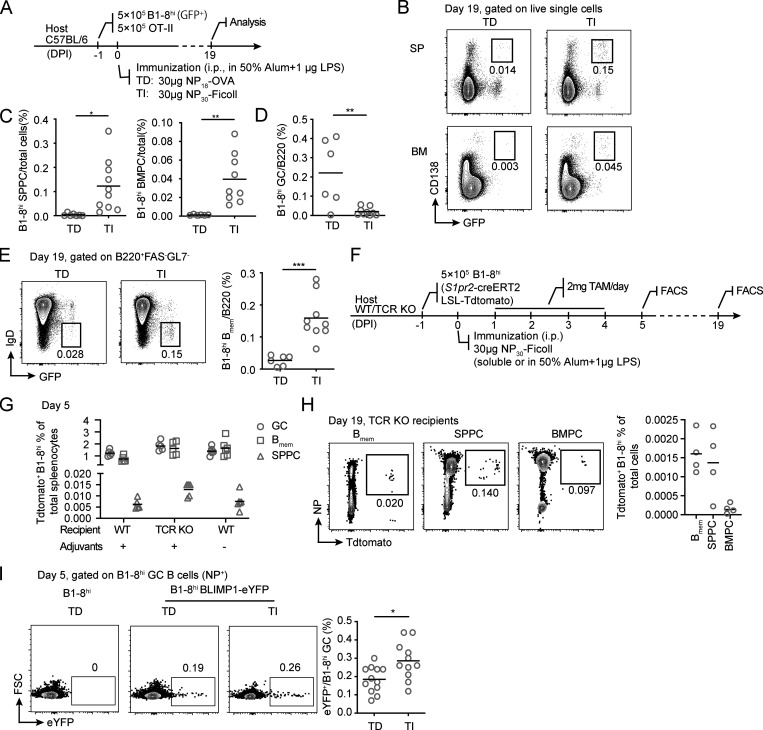
**TI GCs output both B_mem_ cells and PCs. (A–E)** B1-8^hi^ PCs remained in spleen (SP) and bone marrow (BM) 19 d after immunization. **(A)** Experimental outline. **(B)** Representative FACS plots of B1-8^hi^ PC (CD138^+^GFP^+^) in spleen (SPPC) and bone marrow (BMPC). **(C and D)** Summary statistics of percentages of B1-8^hi^ SPPC (C, left), BMPC (C, right), and GC B cells (D). **(E)** Representative FACS plots (left) and summary data (right) showing percentage of GFP^+^ IgD^−^ B1-8^hi^ B_mem_ cells in the spleen of mice 19 d after immunization. **(F–H)** TI GC-derived B1-8^hi^ B_mem_ cells and PCs. **(F)** Experimental outline. TI GCs were constructed using B1-8^hi^ cells carrying the *S1pr2*-creERT2 and Ai14 reporter alleles. **(G)** Quantification of Tdtomato^+^ cells in B1-8^hi^ GC B cells, B_mem_ cells, and SPPCs as a percentage of total splenocytes. **(H)** Representative FACS plots of Tdtomato^+^ B1-8^hi^ cells in splenic B_mem_ cells, SPPCs, and BMPCs (left) and their percentage in total cells (right) 19 d after immunization. **(I)** BLIMP1-eYFP^+^ PC precursors in B1-8^hi^ TD and TI GCs. Representative FACS plots (left) and eYFP^+^ cell percentages (right) are shown. Statistics were pooled from two (A–H) or four (I) independent experiments with at least two mice per group. Each symbol indicates one mouse, and lines denote means. P values by *t* tests. *, P < 0.05; **, P < 0.01; ***, P < 0.001.

To determine whether B_mem_ cells and PCs could develop and exit from TI GCs, we first examined whether these cells could be detected after TI GCs had collapsed (Fig. S1 F). To this end, we induced TI GC and control TD GC formation with B1-8^hi^ cells expressing GFP (Fig. 3 A). 19 d after immunization, GFP^+^ B1-8^hi^ PCs were detected in both the spleen and bone marrow of the hosts immunized with either NP_30_-Ficoll or NP_18_-OVA (Fig. 3 B), while TI GC B cells no longer existed (Fig. 3 D). Interestingly, percentages of GFP^+^ PCs were significantly higher in mice immunized with NP_30_-Ficoll compared with NP_18_-OVA in both spleen and bone marrow (Fig. 3 C). Similarly, more GFP^+^ IgD^−^ B_mem_ cells were detected in the NP_30_-Ficoll immunized animals (Fig. 3 E).

To more stringently verify that those B_mem_ cells and PCs were indeed derived from TI GCs, we used B1-8^hi^ cells that carry the *S1pr2*-creERT2 transgene and the Rosa26-loxP-STOP-loxP-Tdtomato (Ai14) allele, which, in combination, allow for inducible expression of Tdtomato in GC cells and thereby labeling of GC-derived cells (Shinnakasu et al., 2016). After NP_30_-Ficoll immunization, mice were gavaged daily with tamoxifen for a total of 4 d, and percentages of Tdtomato^+^ cells were measured in total B1-8^hi^ GC cells, B_mem_ cells, and PCs on days 5 and 19 after immunization (Fig. 3 F). We confirmed that >70% of B1-8^hi^ GC cells were Tdtomato^+^ at the peak of the response (data not shown). Importantly, Tdtomato^+^ cells were clearly found in B1-8^hi^ B_mem_ and PC compartments, indicating that TI GCs indeed output B_mem_ and PC cells. Furthermore, this output process was not dependent on T cells and did not require adjuvants in the inoculum for immunization (Fig. 3 G). When analyzed 19 d after immunization, Tdtomato^+^ B_mem_ cells and PCs were clearly seen to persist in TCR-knockout hosts (Fig. 3 H), suggesting these TI GC-derived cells can be relatively long-lived. On the other hand, Tdtomato^+^ B_mem_ cells and PCs were not reliably detected in wild-type recipients, most likely as a result of T cell–mediated rejection due to de novo expression of fluorescent protein, a well-noted phenomenon (Bresser et al., 2020; Davey et al., 2013). When BLIMP1-eYFP B1-8^hi^ cells were used as the antigen-specific founder cells, TD GCs contained eYFP^+^ cells, suggesting PC commitment in some GC B cells, as expected according to a previous report (Fooksman et al., 2010). Importantly, a bigger fraction of TI GC B cells were eYFP^+^, further corroborating that TI GC B cells can initiate the PC development program independently of T cell help (Fig. 3 I).

**Figure S2.**
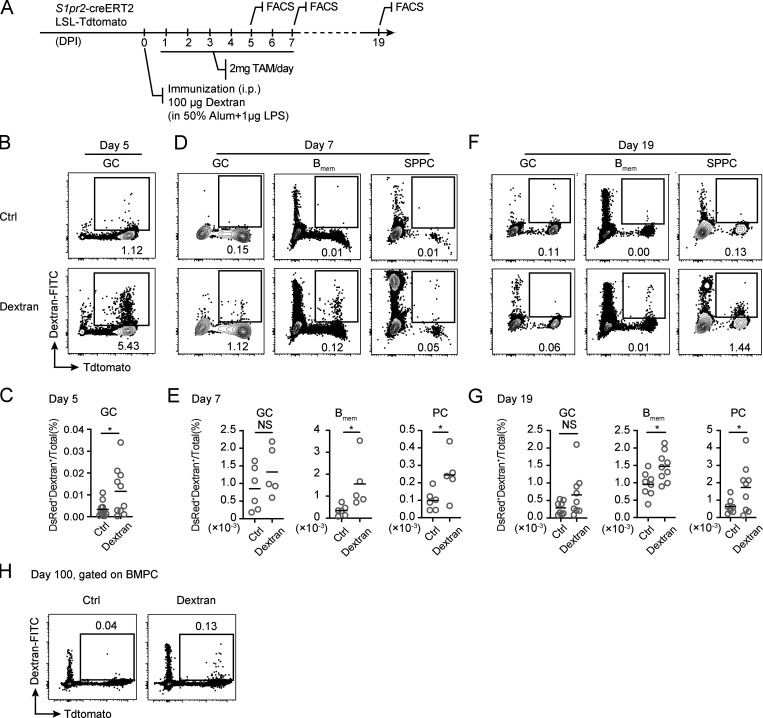
**B_mem_ cells and PCs output from polyclonal TI GCs. (A)** *S1pr2*-creERT2 Ai14 mice were immunized with adjuvant alone or 100 µg dextran with adjuvant and treated with tamoxifen for 7 consecutive days, as shown in the experimental setup. **(B–G)** Percentage of dextran-binding cells in Tdtomato^+^ GC B cells, B_mem_ cells, or SPPCs on days 5, 7, and 19 of the response are shown as FACS plots (B, D, and F) and summary statistics (C, E, and G). Data were pooled from two (E) or three (C and G) independent experiments with at least two mice per group. **(H)** FACS plots showing percentage of dextran-specific Tdtomato^+^ BMPCs on day 100 after immunization. For each experiment, BMPCs were pooled and enriched from four mice. Data represent two independent experiments. In scatter plots, each symbol indicates one mouse, and lines denote means. P values by *t* tests. *, P < 0.05.

To exclude the possibility that B_mem_ and PC output from TI GCs occurs only as a result of adoptive transfer of nonphysiological numbers of transgenic B cells, we also tested dextran immunization in a polyclonal background. *S1pr2*-creERT2 Ai14 mice were immunized with dextran, which is known to induce TI GCs (Wang et al., 1994), and gavaged daily with tamoxifen for a total of 7 d (Fig. S2 A). Consistent with previous findings (Wang et al., 1994), antigen-specific GC B cells were readily detected in the spleen of the animals 5 d after immunization, based on binding to fluorescein-conjugated dextran (Fig. S2, B and C). Similar to the finding in the adoptive transfer model, Tdtomato^+^ dextran-specific B_mem_ cells and PCs were found in immunized mice, but not in control animals injected only with adjuvants, 7 and 19 d after immunization, although Tdtomato^+^ GC cells were no longer detected (Fig. S2, D–G). Furthermore, we were able to detect Tdtomato^+^ dextran-specific bone marrow PCs (BMPCs) 100 d after immunization (Fig. S2 H). Taken together, these results suggest that TI GCs can output relatively long-lived B_mem_ cells and PCs.

### GC experience promotes persistence of B_mem_ cells and PCs in a TI-II response

**Figure 4.**
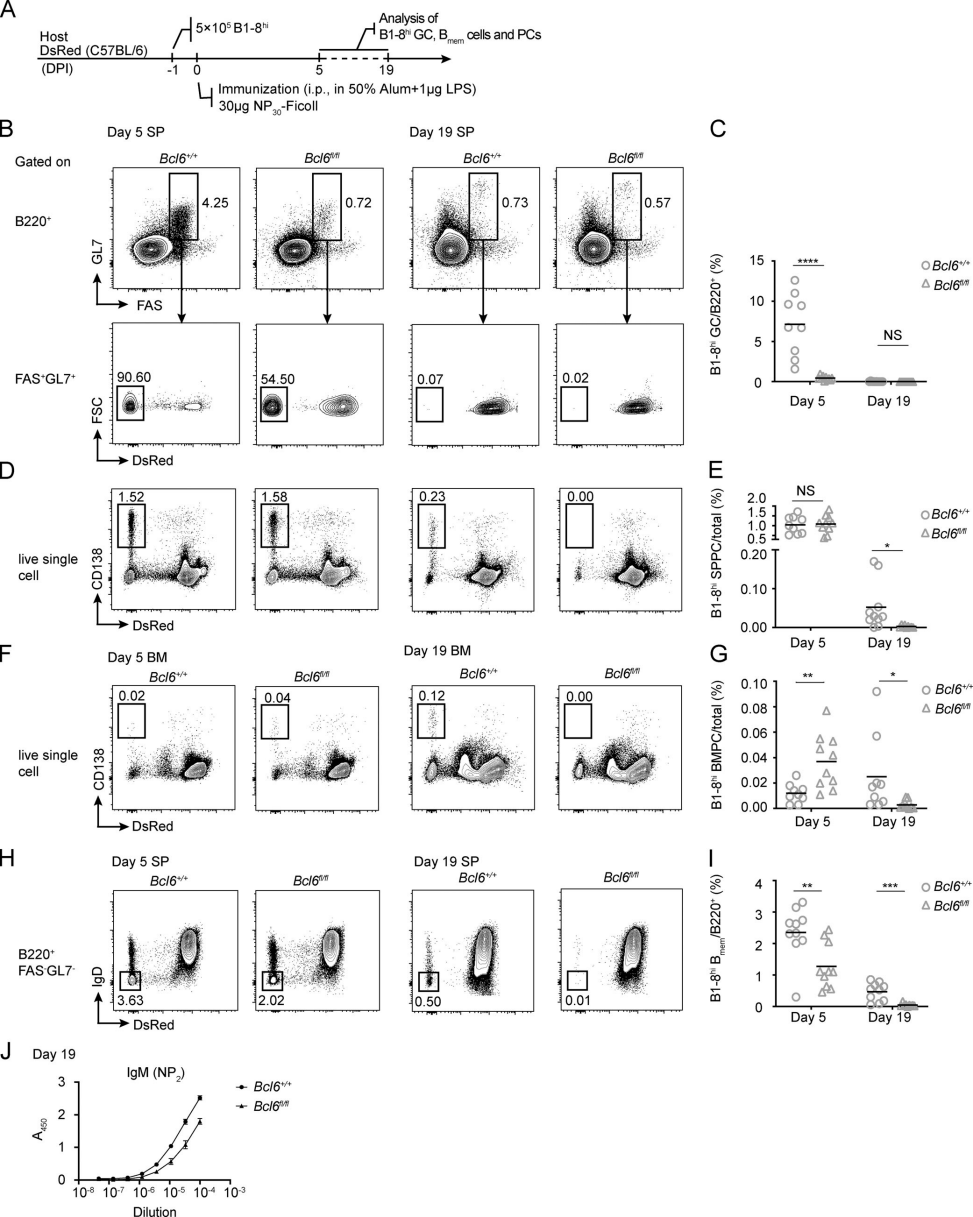
**TI GC formation is required for the induction of immune memory by NP_30_-Ficoll. (A)** B1-8^hi^ cells from *Cd19*^cre/+^*Bcl6^+/+^* or *Cd19*^cre/+^*Bcl6^fl/fl^* mice were transferred into wild-type recipients, and the animals were subsequently immunized i.p. with NP_30_-Ficoll.** (B–I) **FACS plots and summary statistics show the SP GCs (B and C), B1-8^hi^ SPPCs (D and E), B1-8^hi^ BMPCs (F and G) and SP B_mem_ cells (H and I) formed by B1-8^hi^ *Cd19*^cre/+^*Bcl6^fl/fl^* cells compared with wild-type controls on days 5 and 19 after NP_30_-Ficoll immunization, respectively. **(J)** NP-specific IgM antibodies in the serum of mice transferred with either *Cd19*^cre/+^*Bcl6^+/+^* or *Cd19*^cre/+^*Bcl6^fl/fl^* B1-8^hi^ cells, as measured by ELISA. One of two independent experiments with similar results is shown. Data in B–I were pooled from three independent experiments with at least two mice per group. Each symbol in scatter plots indicates one mouse, and lines denote means. P values by *t* tests. *, P < 0.05; **, P < 0.01; ***, P < 0.001; ****, P < 0.0001.

B_mem_ cells and PCs derived from TD GCs are known to persist long after the primary immune response subsides, particularly when compared with PCs generated from extrafollicular responses (Akkaya et al., 2020; Brynjolfsson et al., 2018). We further tested whether the GC program without T cells also promotes persistence of B_mem_ cells and PCs. To this end, we adoptively transferred either *Cd19*^cre/+^*Bcl6^+/+^* or *Cd19*^cre/+^*Bcl6^fl/fl^* B1-8^hi^ cells into congenic recipients and immunized them with NP_30_-Ficoll (Fig. 4 A). Consistent with BCL6 being absolutely required for the establishment of a GC identity (Basso and Dalla-Favera, 2010), few *Cd19*^cre/+^*Bcl6^fl/fl^* B1-8^hi^ cells developed into GCs 5 d after immunization, the peak time point for the response, while by day 19, no GCs remained in either wild-type or BCL6-deficient group (Fig. 4, B and C). Intriguingly, whereas wild-type and BCL6-deficient B1-8^hi^ cells were comparable in generating spleen PCs at the peak time of day 5, PCs in the knockout group precipitously fell and became essentially undetectable at day 19, when many PCs persisted in the wild-type group (Fig. 4, D and E). Moreover, whereas the knockout group had an advantage in producing BMPCs even at day 5, those knockout cells failed to persist to day 19 (Fig. 4, F and G). In line with this clear distinction in persistence, the difference in B_mem_ abundance was ∼2-fold at day 5 but became ∼10-fold by day 19 (Fig. 4, H and I). Consistent with these results, serum levels of anti-NP IgM antibodies were significantly lower in the recipients of BCL6-deficient cells compared with those receiving wild-type cells (Fig. 4 J). Together, these data demonstrate that TI-II antigen can induce GCs that export B_mem_ and PCs and suggest that even a brief experience of the GC stage in a TI response encourages longer persistence of PCs and B_mem_ cells.

### Infant B cells have a reduced ability to form TI GCs

**Figure 5.**
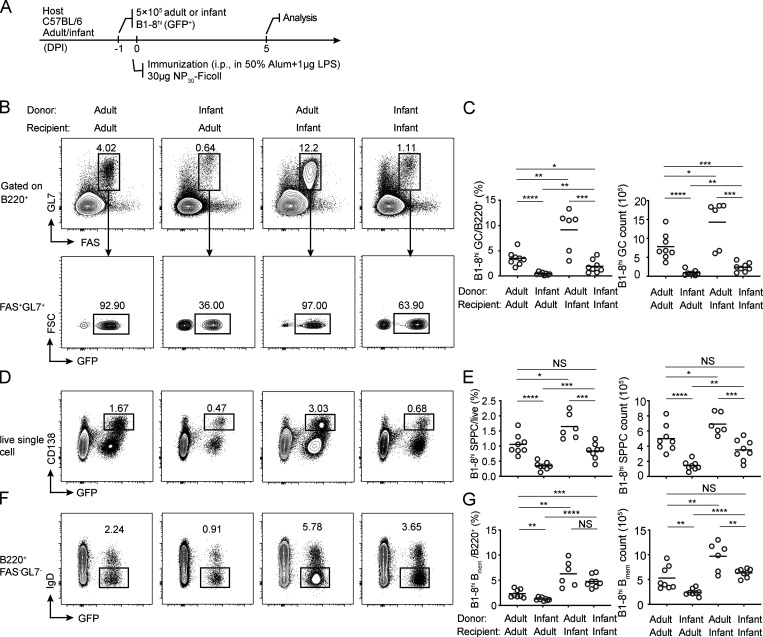
**Infant B cells are intrinsically unable to form TI GCs. (A)** The protocol. B1-8^hi^ GFP^+^ cells from adult (8–12 wk) or infant (3 wk) mice were transferred into either adult or infant recipients as indicated, and TI GCs were constructed by i.p. NP_30_-Ficoll immunization. **(B–G)** FACS dot plots and summary statistics show TI GCs (B and C), PCs (D and E), and B_mem_ cells (F and G) in the spleen of recipients, 5 d after immunization. Data were pooled from two independent experiments with at least three mice per group. Each symbol indicates one mouse, and lines denote means. P values by *t* tests. *, P < 0.05; **, P < 0.01; ***, P < 0.001; ****, P < 0.0001.

Children <2 yr old are most susceptible to infections by encapsulated bacteria and respond poorly to polysaccharide vaccines, because of their apparent failure to generate functional memory against these antigens, but the underlying reason is not fully understood (Siegrist and Aspinall, 2009). Infant animals also respond poorly to TI-II antigen immunization (Shriner et al., 2010). Given our observation that GC formation promotes persisting memory formation following TI-II antigen challenge, we explored whether infants and adults are different in the ability to mount TI GCs. We transferred B1-8^hi^ cells from either infant (3-wk-old) or adult (6–10-wk-old) donor mice into infant or adult B6 recipients and examined TI GC formation after NP_30_-Ficoll immunization (Fig. 5 A). As expected, adult B1-8^hi^ cells formed GCs and generated PCs and B_mem_ cells robustly in adult hosts. However, an equal number of input infant B1-8^hi^ cells were only able to generate ∼5% as large a GC response as their adult counterparts (Fig. 5, B and C). On the other hand, in the infant host, adult B1-8^hi^ cells gave rise to more GC B cells than in the adult host (Fig. 5, B and C). Therefore, there is an intrinsic inability on the part of infant B cells, but not of the infant host environment, to generate TI GCs. Consistent with this B cell–intrinsic effect of age, infant B cells also produced fewer PCs (Fig. 5, D and E) and fewer B_mem_ cells in either adult or infant hosts (Fig. 5, F and G). This infant defect could contribute to the apparent lack of humoral memory in response to TI-II antigens.

**Figure S3.**
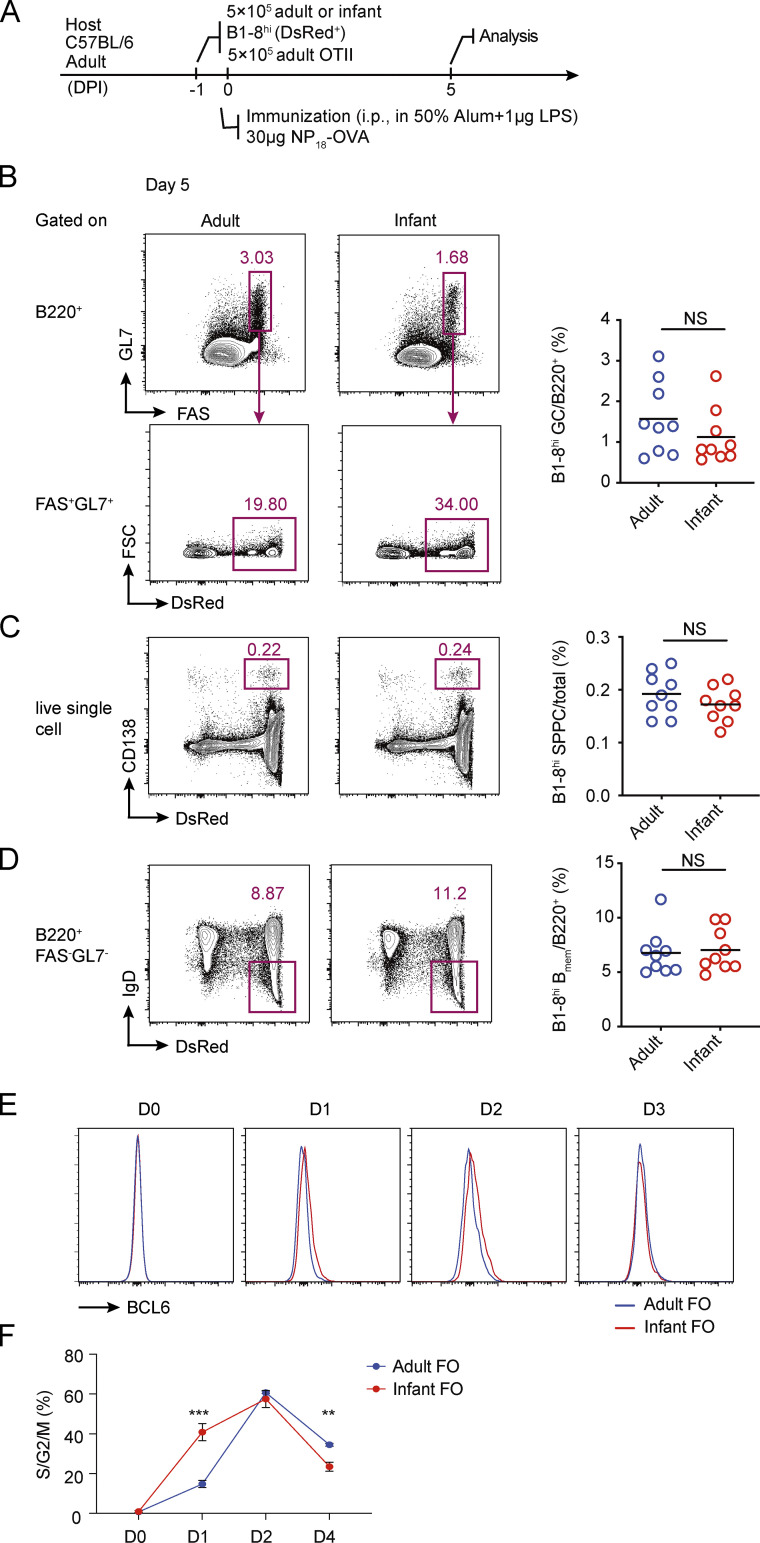
**TD GC formation by adult and infant B1-8^hi^ B cells. (A–D)** A total of 5 × 10^5^ B1-8^hi^ DsRed^+^ B cells from either adult (8–12 wk) or infant (3 wk) mice were transferred into congenic adult mice with adult OT II cells, and the recipients were immunized i.p. with NP_18_-OVA to construct TD GCs. **(A)** Experimental setup. **(B–D)** FACS plots (left) and summary statistics (right) show the percentages of B1-8^hi^ GC cells (B), PCs (C), and B_mem_ cells (D) in the spleen 5 d after immunization. Data were pooled from three independent experiments with at least two mice per group. Each symbol indicates one mouse, and lines denote means. P values were calculated using student *t* test. **(E and F)** Follicular B cells (CD19^+^CD21^hi^CD23^lo^) were sorted from either adult or infant mice and cultured in vitro according to the Nojima protocol. **(E)** FACS data showing BCL6 up-regulation at different time points after cell activation. **(F)** Summary data showing percentages of cells in S/G2/M phase of cell cycle at different time points, as measured by 7-AAD staining. Error bars denote SD. Data are representative of two independent experiments, with three mice analyzed per group. P values by *t* tests. **, P < 0.01; ***, P < 0.001.

Although infants respond poorly to polysaccharide vaccines, it is known that they could establish immune memory against polysaccharide antigens conjugated to protein carriers, although still not as efficiently as adults (Adkins et al., 2004). Therefore, we tested whether infant B1-8^hi^ cells were capable of forming TD GCs. To do that, we adoptively transferred either adult or infant B1-8^hi^ cells into adult recipients together with adult OT-II cells, and recipient mice were i.p. immunized with NP_18_-OVA (Fig. S3 A). 5 d after immunization, GC formation by infant B1-8^hi^ cells was comparable to that of adult B1-8^hi^ cells (Fig. S3 B). Moreover, the generation of B1-8^hi^ B_mem_ cells and PCs was not affected (Fig. S3, C and D). Therefore, infant B cells are severely limited in GC participation when T cell help is not available. To test whether a high level of T cell help can indeed revert this limitation, we compared infant and adult B cells in vitro using the Nojima culture protocol, which constantly provides B cells with CD40L and B cell–activating factor and thereby mimics strong T cell help (Nojima et al., 2011). Interestingly, under this nonlimiting help condition, naive infant B cells not only acquired GC markers sooner than adult counterparts (Fig. S3 E) but also entered the cell cycle faster (Fig. S3 F).

## Concluding remarks

TI-II antigens are known to induce relatively long-lived PC differentiation in experimental animals (Bortnick et al., 2012; Foote et al., 2012; Taillardet et al., 2009), and vaccines containing only TI-II antigens provide ≤5 yr of protection in human adults (Robbins et al., 1983), but it has not been clear how humoral immune memory could be generated without the involvement of T cells. We now report that the transient GC formation induced by TI-II antigens is productive in generating both B_mem_ cells and PCs. By using genetic models that prevent GC formation in a B cell–intrinsic manner, we also show that progression through a GC stage, albeit brief, facilitates immune memory formation. Our findings suggest a functional link between TI GC formation and TI humoral memory that would underlie vaccine protection.

Our conclusions seemingly contradict the initial report of TI GCs (de Vinuesa et al., 2000), in which the authors considered TI GCs as transient structures terminated by massive apoptosis. However, consistent with our cytometric quantitation with caspase staining, the earlier report documented only a small fraction of apoptotic cells in GCs by terminal deoxynucleotidyl transferase dUTP nick-end labeling (TUNEL) staining (de Vinuesa et al., 2000), at a level comparable to what was observed in TD GCs that last for ≥2–3 wk (Mayer et al., 2017). Moreover, the initial report also documented a considerable number of NP-reactive cells on splenic tissue sections after the collapse of TI GCs (de Vinuesa et al., 2000), making it possible that some of those cells might be GC emigrants as well. Nonetheless, the fact that TI GCs output both PCs and B_mem_ cells is actually consistent with the fact that the internal machinery for GC formation, memory, and particularly PC differentiation can be turned on in B cells by antigen receptor signaling alone (Radtke and Bannard, 2019; Shinnakasu et al., 2016; Zhang et al., 2017). It appears that, in the case of TD GCs, the global effect of T cell help is not to trigger the onset or instruct the progression of a humoral response but to amplify and sustain what B cells are already capable of by themselves. Given the severe depletion of cells of the LZ phenotype in TI GCs, arguably the main and essential effect of T cell help in TD GCs is to positively select LZ cells for cyclic reentry into the DZ and maintenance of the GC reaction. Alternatively, the extremely strong antigen receptor cross-linking induced by TI-II antigen may lead to a strong tendency of rapid terminal differentiation, which would not happen in TD GCs.

Taking advantage of the fact that BCL6 is absolutely required for GC development, we show that the longevity of both B_mem_ cells and PCs induced as a result of TI antigen immunization is significantly prolonged when TI GCs can form. Although we cannot fully rule out the possibility that BCL6 expressed at the pre-GC stage (Robinson et al., 2020) contributes to the persistence of these cells, it is unlikely that BCL6 functions only at that stage but not in GCs. In fact, our observation echoes the findings from TD responses in that TD GC–derived memory and PCs have a unique advantage to persist longer (Akkaya et al., 2020; Brynjolfsson et al., 2018). The long-term survival of PCs and B_mem_ cells is determined by factors including epigenetic remodeling and establishment of survival niches (Khodadadi et al., 2019; Zan and Casali, 2015). It is known that GC B cells undergo profound remodeling, both transcriptionally and epigenetically. For example, GC B cells highly express a number of epigenetic modulators, such as AID, NSD2, DNMT1, EZH2, and UHRF1, which may affect the differentiation of both B_mem_ cells and PCs (Béguelin et al., 2017; Chen et al., 2018a; Dominguez et al., 2015; Good-Jacobson, 2014; Guo et al., 2018; Shaknovich et al., 2011). For example, EZH2 is known to regulate PC maintenance and function in murine models (Guo et al., 2018), and the abundance of PCs and B_mem_ cells is greatly reduced in NSD2-deficient animals (Chen et al., 2018b). In light of our results, it will be interesting to consider whether GC-specific reprogramming events would imprint longevity features into post-GC memory and PCs.

The proportion of PCs derived from *S1pr2*-creERT2–expressing TI GC precursors is relatively low, compared with total PCs generated during the response (Figs. 3 H and S2 B). Several aspects need to be considered. Due to incomplete labeling of GC-experienced cells, the measured abundance of TI GC–derived PCs based on Tdtomato expression is likely to be an underestimate. More importantly, the rarity of Tdtomato^+^ PCs shown in these experiments is not incompatible with a contribution of TI GC–derived PCs to a persisting PC compartment. First of all, intrinsic properties of PCs, rather than the number, likely determine their persistence. For example, in experiments described in Fig. 4, we found that although wild-type and BCL6-deficient B1-8^hi^ cells differentiated into comparable numbers of spleen PCs (SPPCs) and BMPCs on day 5 after immunization, significantly fewer BCL6-deficient PCs persisted until day 19. Second, long-lived PCs are indeed very rare. In humans, it is estimated that long-lived PCs specific to a given vaccine take up <0.5% of total IgG BMPCs (Halliley et al., 2015). Third, the mode of antigen depositions determined by different immunization routes (Song and Cerny, 2003) likely contribute to the extent of extrafollicular responses. Given that the polysaccharide vaccines are normally given intramuscularly rather than intraperitoneally, the relative contribution of GC-dependent and -independent immune memory in humans following vaccination remains to be fully evaluated.

Compared with adults, human infants have an impaired ability to generate humoral memory, especially in response to vaccines composed of TI-II antigens (Siegrist and Aspinall, 2009). These defects in part explain infant susceptibility to encapsulated bacterial infections and can be recapitulated in murine models (Shriner et al., 2010). However, these defects do not seem to result from general impairment of the proximal antigen-receptor signaling, because infant B cells are as sensitive to antigen receptor stimulation as adult cells (Glaesener et al., 2018; Tasker and Marshall-Clarke, 2003). Instead, it generally has been believed that the defect results from the lack of marginal zone B cells in infants (Siegrist and Aspinall, 2009). In light of our findings that murine infant B cells do not efficiently form GCs or produce memory and PCs in response to TI-II antigen, we speculate that a specific mechanism in B cells inhibits GC development from infant B cells, which can be alleviated with ample T cell help, and contributes to the lack of humoral memory formation against TI-II antigens in young animals. Definition of this putative mechanism may hold the key to formulate better protein-free polysaccharide vaccines for infants.

## Materials and methods

### Mice

BLIMP1-eYFP (JAX 8828), dsRed-expressing (JAX 6051), T cell receptor transgenic OT-II mice (JAX 4194), and the Ai14 reporter (JAX) were from the Jackson Laboratory. B1-8^hi^ mice expressing the photo-activatable GFP (Victora et al., 2010), *S1pr2*-creERT2 mice (Shinnakasu et al., 2016), and UBP-2A-FUCCI mice (Wang et al., 2017) were as previously described. All mice were maintained under specific pathogen–free conditions. Animal experiments were performed according to governmental and institutional guidelines for animal welfare and approved by the institutional animal care and use committee at the Tsinghua University.

### Antibodies and chemicals

Fluorophore-labeled anti-B220 (RA3-6B2) and anti-FAS (Jo2) antibodies were from BD; anti-CD138 (281-2), anti-CD21/35 (CR2/CR1), and anti-IgD (11-26c2a) antibodies were from BioLegend; anti-GL7 (GL7), anti-CD38 (90), and anti-rabbit IgG antibodies were from Invitrogen; rabbit anti-cCasp3 and the matching isotype control antibodies were from Cell Signaling; and anti-GFP antibody was from Abcam. The fixable viability dye, zombie yellow, was from BioLegend. NP_18_-OVA, NP_30_-Ficoll, NP_2_-BSA, NP-PE, and 4-hydroxy-3-iodo-5-nitrophenylacetyl (NIP)-BSA-biotin were from Biosearch Technologies. NP-APC conjugates were made by allowing NP-Osu (Biosearch Technologies) and APC (BioLegend) to react for 4 h at room temperature in a buffer containing 0.1 M NaHCO_3_ and 0.15 M NaCl_2_ (pH 8), at a molar ratio of 20:1. Dextran (200 kD, 31398) and dextran-FITC (2,000 kD, FD2000S) were from Sigma-Aldrich.

### Cell isolation

B cells and T cells were negatively and positively isolated from spleens of B1-8^hi^ and OT-II mice through the use of CD43 and CD4 microbeads (Miltenyi Biotec), respectively. The number of NP-binding B1-8^hi^ cells was quantified by flow cytometry.

### Immunization

To construct TD and TI GCs, a total of 5 × 10^5^ B1-8^hi^ B cells and 5 × 10^5^ OT-II T cells were transferred into each congenic recipient, followed by i.p. immunization with 30 µg of either NP_(18)_-OVA or NP_(30)_-Ficoll. The immunogen was emulsified in 50% alum (Thermo Fisher Scientific) containing 1 µg LPS (Sigma-Aldrich). In experiments examining TI GCs alone, only B1-8^hi^ B cells were transferred. For the analysis of dextran-specific GC B cells and GC-derived cells, each *S1pr2*-creERT2 Ai14 mouse was immunized i.p. with 100 µg dextran emulsified in 50% alum containing 1 µg LPS.

### Flow cytometry analysis of GC B cells, PCs, and B_mem_ cells

Splenocytes and bone marrow cells were stained with fluorescently labeled antibodies, and different subsets of cells were gated as shown in Fig. S1 F. Cells derived from the transferred B1-8^hi^ cells were further gated based on their different fluorescent protein expression from the host or their binding to NP or NIP. In polyclonal response against dextran, splenocytes and bone marrow cells were stained with 10 µg/ml dextran-FITC to identify antigen-specific cells in each population. Intracellular staining were used to identified NP- or dextran-specific PCs at later time points (days 7, 19, and 100) of the responses, as these cells have lost their surface BCR expression. Data were acquired using an Aria III or LSRII flow cytometer (BD) or an Aurora cytometer (Cytek).

### Apoptosis assay

Cells were stained with various antibodies against surface antigens together with zombie yellow, fixed, permeabilized (Cytofix/Cytoperm kit; BD), and stained with a rabbit anti-cCasp3 antibody or isotype control followed by AF647-conjugated secondary antibody. Apoptotic cells were gated as zombie yellow^−^cCasp^+^.

### BrdU assay

30 min before the experiment, 2 mg BrdU was injected i.v. into each mouse. Splenocytes were stained with antibodies against surface antigens, fixed, and permeabilized, and intracellular BrdU was stained (BrdU staining kit; BD) according to the manufacturer’s instructions.

### Immunohistochemistry

Sections of spleens were fixed, dehydrated, and frozen as previously described (Bajénoff et al., 2003). Frozen spleens were sliced into 18-µm thin sections and stained with IgD-Pacific Blue and CD21/35-APC antibodies to visualize B cell follicles and follicular dendritic cells, respectively. Transferred B1-8^hi^ GFP^+^ cells were stained with a rabbit anti-GFP antibody (ab6556; Abcam) followed by a secondary antibody conjugated with AF488 (Invitrogen). Images were captured using an Olympus FV1000 upright microscope. The areas of DZ and LZ within GCs were quantified by ImageJ (National Institutes of Health).

### Cell cycle analysis of B1-8^hi^ GC B cells

For cell cycle analysis based on DNA contents, cells were stained with different antibodies against surface antigens, fixed, permeabilized (Foxp3 fixation kit; Thermo Fisher), and stained with DAPI (1 µg/ml, Sigma-Aldrich) and antibodies against Ki67 (Ki67 staining kit; BD) according to the manufacturer’s instructions. B1-8^hi^ cells were identified through their binding to NP-PE or NIP-BSA-biotin as indicated.

### Tamoxifen induction of Tdtomato expression in vivo

After immunization, mice were gavaged daily with 2 mg of tamoxifen dissolved in 200 µl of sunflower seed oil (Sigma-Aldrich) for 4 or 7 d. Splenocytes and bone marrow cells were analyzed for Tdtomato expression at different time points after immunization as indicated.

### ELISA

Plates were coated with 0.5 µg NP_2_-BSA at 5 µg/ml and 4°C overnight and washed three times with washing buffer (PBS containing 0.05% Tween 20). Subsequently, the plates were blocked by 5% BSA in washing buffer for 1 h at room temperature. Serums were diluted in washing buffer containing 1% BSA, added to the plates, and incubated for 2 h at room temperature. The plates were washed three times and incubated with anti-IgM-HRP antibody (ab97230; Abcam) for 2 h at room temperature. Excess secondary antibodies were washed off, and the plates were developed by the addition of TMB substrates (BioLegend) according to the manufacturer’s instructions.

### Online supplemental material

Fig. S1 shows DZ LZ polarization in TI GCs as well as the gating strategies for GCs, B_mem_ cells, and PCs. Fig. S2 shows the B_mem_ cells and PCs output from polyclonal TI GCs at various time points after dextran immunization. Fig. S3 shows TD GC formation by adult and infant B1-8^hi^ B cells.
